# An ecological analysis of food outlet density and prevalence of type II diabetes in South Carolina counties

**DOI:** 10.1186/s12889-015-2681-6

**Published:** 2016-01-05

**Authors:** Dana M. AlHasan, Jan Marie Eberth

**Affiliations:** 1 Department of Epidemiology and Biostatistics, University of South Carolina, Columbia, SC USA; 2 Department of Epidemiology and Biostatistics, Statewide Cancer Prevention and Control Program, University of South Carolina, Columbia, SC USA

**Keywords:** South Carolina, Geographic information systems, Diabetes, Fast food restaurants, Convenience stores, Super stores, Grocery stores

## Abstract

**Background:**

Studies suggest that the built environment with high numbers of fast food restaurants and convenience stores and low numbers of super stores and grocery stores are related to obesity, type II diabetes mellitus, and other chronic diseases. Since few studies assess these relationships at the county level, we aim to examine fast food restaurant density, convenience store density, super store density, and grocery store density and prevalence of type II diabetes among counties in South Carolina.

**Methods:**

Pearson’s correlation between four types of food outlet densities- fast food restaurants, convenience stores, super stores, and grocery stores- and prevalence of type II diabetes were computed. The relationship between each of these food outlet densities were mapped with prevalence of type II diabetes, and OLS regression analysis was completed adjusting for county-level rates of obesity, physical inactivity, density of recreation facilities, unemployment, households with no car and limited access to stores, education, and race.

**Results:**

We showed a significant, negative relationship between fast food restaurant density and prevalence of type II diabetes, and a significant, positive relationship between convenience store density and prevalence of type II diabetes. In adjusted analysis, the food outlet densities (of any type) was not associated with prevalence of type II diabetes.

**Conclusions:**

This ecological analysis showed no associations between fast food restaurants, convenience stores, super stores, or grocery stores densities and the prevalence of type II diabetes. Consideration of environmental, social, and cultural determinants, as well as individual behaviors is needed in future research.

**Electronic supplementary material:**

The online version of this article (doi:10.1186/s12889-015-2681-6) contains supplementary material, which is available to authorized users.

## Background

Type II diabetes mellitus (DM) is growing at an alarming rate worldwide and in the United States (U.S.). An estimated 221 million adults in the world [1], and an estimated 25.8 million people in the U.S. have DM [2]. In South Carolina, one of 15 states that compromises the diabetes belt [3], an estimated 450,000 individuals are diagnosed with DM, which does not include those undiagnosed or with pre-diabetes [4]. In fact, DM is the 7^th^ leading cause of death in South Carolina [5], and this state ranks as the 4^th^ highest in the nation in terms of DM prevalence [5].

When DM is not managed, it leads to complications including but not limited to cardiovascular diseases, retinopathy, and kidney failure [6, 7]. These complications increase risk for disability, risk for mortality, hospitalization rates, and medical costs thus placing additional physical and financial burdens upon the individual [8]. Likewise, healthcare institutions treating DM complications take on financial burdens [8, 9]. In 2012, the total estimated costs in the U.S. from DM were $245 billion [10]. Similarly, in 2013, the total amount for hospital charges related to DM diagnosis in South Carolina was $321 million [5]. Hence, it is essential to understand DM risk factors and to design effective interventions to reduce the risks and costs associated with DM treatment.

Known risk factors of DM include genetic predisposition, age (≥45 years), obesity, physical inactivity, and diet- particularly foods rich in carbohydrates [8, 11]. Race or ethnic background is also a significant predictor of DM. DM disproportionately affects non-Hispanic Blacks compared with Whites [11, 12]. In 2011, an estimated 12.6 % of non-Hispanic Black adults (age ≥ 20) were diagnosed with DM [2], and in 2011, 1 in 8 African Americans in South Carolina have DM [5].

Unfortunately, many weight loss interventions geared towards individuals have been unsuccessful in reducing DM prevalence [13]. Therefore, it is essential for research to focus on the built environment in which individuals’ behavioral decisions are influenced [14]. Studies show the food environment, especially those with a high number of fast food restaurants and convenience stores, to be associated with increased dietary intake [15, 16], and that exposure to poor food quality has important effects on overweight and obesity [17, 18]. Such food environments are defined as obesogenic environments because they have high levels of nutrient-deficient, highly-caloric, affordable food that promote food consumption and physical inactivity [13, 19].

Fast food restaurants particularly provide foods lacking in nutritional value (i.e. foods low in calcium, folate, vitamin A, vitamin C, and dietary fiber) and thus contribute to poor quality dietary patterns [20]. Davis et al. demonstrated that students whose schools were proximal to fast food restaurants on average consumed more servings of soda and were more overweight compared to students whose schools were less proximal to fast food restaurants [17]. Babey et al. showed that people in communities with increased fast food restaurants and less grocery stores are more likely to have DM [21].

Similar to fast food restaurants, convenience stores are stocked with low quality food options. Galvez et al. illustrated that the presence of convenience stores is associated with increased risk of obesity [22]. Because obesity is a predictor of DM, it is hypothesized that the presence of convenience stores will also be associated with DM.

It is also suggested that lack of supermarkets and grocery stores, which provide healthy options, contribute to the risk of obesity [23]. Inagami’s et al. study revealed that abundance of and proximity to supermarkets within residential census tracts were associated with increased fruit and vegetable intake among pregnant women [24]. More so, distance from the home to a super or grocery store may limit accessibility because some individuals may live farther away or lack the transportation to obtain high quality foods [11].

Few studies have assessed the association between food environment and DM at the macro level. Yet, it is important for county-level studies to research how attributes within the built environment contribute to DM prevalence and research how access to food influences eating behavior [25]. Understanding the role food outlets play in contributing to DM is vital because it can greatly contribute to future public policy making. Thus, further efforts to understand how the built environment plays a role among the known DM risk factors are needed. In particular, how do types and amount of food outlets play a role in DM prevalence?

This study aims to examine the relationship between the density of four types of food outlets- fast food restaurants, convenience stores, super stores, and grocery stores- and the prevalence of county-level DM in 2011 in South Carolina, while adjusting for the following county-level risk factors: obesity, physical inactivity, recreation facility density, unemployment, education, households with no cars and limited access to stores, and race.

## Methods

### Study design

This study was an ecological analysis examining the relationship between four types of food outlet densities- fast food restaurants, convenience stores, super stores, and grocery stores- and DM prevalence in 2011 among the 46 counties of South Carolina. The analysis controlled for the following county-level covariates: obesity, physical inactivity, density of recreation facilities, unemployment, education, households with no car and limited access to stores, and race. Multiple secondary data sources were used: the US Census Topologically Integrated Geographic Encoding and Referencing (TIGER) Line Files [26], the Centers for Disease Control and Prevention (CDC) which administers the Behavioral Risk Factor Surveillance System (BRFSS) [27], the US Department of Agriculture Economic Research Service (USDA) [28], and the US Decennial Census [29]. All of the data used was publically available.

### Data collection

The South Carolina county map was retrieved from the US Census TIGER Line Files for 2011 [26]. County polygons were joined with county-level covariates using the “spatial join” tool in ArcGIS Desktop Version 10.2.2 for Windows (Environmental Systems Research Institute, Redlands, CA).

#### Dependent variable: DM

DM prevalence is the age-adjusted percentage of adults (age ≥ 20) in South Carolina with diabetes excluding those with gestational diabetes in 2011. DM prevalence was obtained from the CDC [27].

#### Independent variables

Fast food restaurants, convenience stores, super stores, and grocery stores were downloaded from the USDA 2011 data [28]. Because urban areas are correlated with neighborhood availability of food outlets and individual eating behavior [30], population density is taken into account by dividing the number of food outlets per 1000 county residents.

Fast food restaurant densities are the number of limited-service restaurants in each county in South Carolina per 1000 residents. The North American Industry Classification System (NAICS) code for fast food restaurants is 722211 and includes businesses mainly engaged in providing food services where customers pay before eating. Food may be consumed at the establishment, taken out, or delivered.

Convenience store densities are the number of convenience stores in each county in South Carolina per 1000 residents. The NAICS codes for convenience stores are 445120 and 447110 and includes stores mainly engaged in providing limited goods such as bread, soda, and snacks.

Super store densities are the number of supercenters and warehouse clubs in each county in South Carolina per 1000 residents. The NAICS code for super stores is 452910 and includes stores mainly engaged in providing groceries along with general lines of merchandise.

Grocery store densities are the number of supermarkets and grocery stores in each county in South Carolina per 1000 residents. The NAICS code for grocery store is 445110 and includes stores mainly known as supermarkets and excludes convenience stores with or without gasoline sales as well as large, general-merchandise stores.

#### Covariates

The covariates considered for this study are obesity, physical inactivity, recreation, unemployment, education, households with no car and at least one mile away from a store, and race.

Obesity prevalence is the age-adjusted percentage of adults (age ≥ 20) in South Carolina with a body mass index ≥ 30 in 2011. Body mass index is computed by dividing weight in kilograms by height in meters squared. Physical inactivity prevalence is the age-adjusted prevalence of adults (age ≥ 20) in South Carolina, who reported no leisure-time physical activity in the past 30 days in 2011. These data were obtained from the CDC collected from BRFSS [27], which is based on self- report measures.

Recreation is the number of recreational facilities in each county in South Carolina per 1000 residents in 2011 and obtained from the USDA [28]. The NAICS code is 713940 and includes establishments mainly engaged in fitness and sport facilities.

Unemployment is the unemployment rate in each county in South Carolina in 2011. This was downloaded from the US Census 2010 [29] that is collected from the Bureau of Labor Statistics. Education is the percentage of adults with only a high school diploma in each county in South Carolina. These data were also downloaded from the US Census, specifically the 2009-2013 American Community Survey.

Households with no cars and at least one mile from a store is the percentage of housing units in each county in South Carolina with no car and at least one mile away from a supermarket or large grocery store in 2010. These data were obtained from the USDA [28].

Race is the percentage of each county’s resident population in South Carolina that is non-Hispanic Black or African American in 2010 obtained from the USDA [28]. Neither of these data were available for the year 2011. A description of the data sources used to extract these variables is found in Additional file 1.

### Data analysis

Pearson’s correlation coefficients were computed to assess the crude relationship between each food outlet density and DM prevalence in SAS software, Version 9.3 for Windows (SAS Institute, Cary, NC). Bivariate maps were created in ESRI ArcGIS (Environmental Systems Research Institute, Redlands, CA) to represent the geographical distribution of food outlet densities and DM prevalence among the 46 counties in South Carolina. DM prevalence was split into three ranks based on the following measures: 7.6–10.5 %, 10.6–13.0 %, and 13.1–15.6 %. Likewise, food outlet densities were split into three ranks. Fast food restaurant densities were based on the following categories: 0.25–0.50, 0.51–0.70, and 0.71–1.13. Convenience store densities: 0.32–0.60, 0.61–0.80, and 0.81–1.12. Super store densities: 0–0.001, 0.002–0.02, and 0.03–0.04. Grocery store densities: 0–0.15, 0.16–0.20, and 0.21–0.31. Categories were created based on equal intervals or included at least ten counties in a category.

To analyze the association between each food outlet density and DM prevalence, linear regression models using ordinary least squares (OLS) method were calculated in ArcGIS. OLS regression was completed with DM as the dependent variable and adjusting for obesity, physical inactivity, recreation, unemployment, education, households with no car and limited access to a store, and race. Diagnostics of the models were performed and did not show spatial autocorrelation and did not violate other model assumptions. Multicollinearity was assessed and all Variance Inflation Factors were less than 7.0. Diagnostics also showed that the variables explain 80 % of the variation observed.

## Results

### Descriptive statistics

Table 1 summarizes mean values of DM prevalence, food outlet densities, and covariates for each county in South Carolina. The maximum value of DM prevalence was 15.6 % (Hampton County), the minimum was 7.6 % (Beaufort County), and the overall mean prevalence of DM was 12.1 %. The maximum value of fast food restaurant densities was 1.14 (Horry County), the minimum was 0.25 (Saluda County), and the overall state mean was 0.59. The maximum value of convenience store densities was 1.12 (Marlboro County), the minimum was 0.32 (Berkeley County), and the overall state mean was 0.69. The maximum value of super store densities was 0.04 (Barnwell County), the minimum was 0 (several counties), and the overall state mean was 0.01. The maximum value of grocery store densities was 0.31 (Bamberg County), the minimum was 0.07 (Calhoun County), and the overall state mean was 0.19. There was also variation among the covariates between counties (see Table 1).

**Table 1:** Descriptive characteristics of Counties in South Carolina

County	Density of fast food restaurants	Density of convenience stores	Density of super stores	Density of grocery stores	Diabetes prevalence (%)	Obesity prevalence (%)	Physical inactivity prevalence (%)	Density of recreation facilities	Densities of households with no car and greater than 1 mile from store	Unemployment rate	Education	Non-hispanic black or African American (%)
Abbeville	0.397	0.477	0	0.199	11.4	34.8	28.9	0	6.05	12.4	33.41	28.17
Aiken	0.548	0.573	0.019	0.180	10.1	31.4	24.9	0.06	3.24	9.2	31.57	24.39
Allendale	0.393	0.786	0	0.196	15.4	39.4	33.7	0	4.08	18.4	34.12	73.38
Anderson	0.817	0.690	0.016	0.149	10.6	33.4	28.8	0.06	3.26	10.3	31.91	15.93
Bamberg	0.626	0.626	0	0.313	14.4	41.1	31.6	0.06	3.97	16.2	26.02	61.21
Barnwell	0.537	0.850	0.045	0.179	11.9	38.3	28.9	0.04	6.38	15.2	39.05	44.05
Beaufort	0.656	0.383	0.012	0.200	7.6	22.5	16	0.16	3.05	8.7	24.37	18.90
Berkeley	0.605	0.321	0.016	0.104	12.7	36.3	25.6	0.05	2.56	9.5	31.69	24.75
Calhoun	0.264	0.792	0	0.066	14.9	39.7	28.7	0	8.33	12.3	37.77	42.31
Charleston	0.987	0.461	0.020	0.274	10.4	25.9	21.1	0.11	2.26	8.3	21.30	29.55
Cherokee	0.702	0.774	0.018	0.180	11.5	32.8	32.2	0.07	3.49	14.7	35.15	20.26
Chester	0.456	0.790	0	0.304	12.3	37.2	27.2	0.06	5.18	17.3	38.83	37.26
Chesterfield	0.473	0.558	0.021	0.258	11.9	35.4	30.3	0.04	7.26	12.9	38.65	32.48
Clarendon	0.605	1.037	0.029	0.115	13.5	38.3	28	0.03	5.29	14.5	38.06	49.70
Colleton	0.647	0.932	0.026	0.285	14.2	38.2	30.5	0.05	7.63	14.1	35.39	38.85
Darlington	0.454	0.805	0.015	0.190	13.5	33.8	32	0.09	3.81	12.9	36.89	41.44
Dillon	0.567	0.850	0	0.252	13.2	39.8	35.9	0	7.26	15.8	39.60	45.94
Dorchester	0.539	0.341	0.007	0.121	9.7	30	22.9	0.05	2.36	8.9	29.31	25.53
Edgefield	0.300	0.450	0	0.112	11.6	33.5	26	0.04	4.12	9.9	37.95	36.88
Fairfield	0.297	0.764	0.042	0.085	15.3	40.6	29.7	0	6.26	14.5	39.57	58.91
Florence	0.791	0.696	0.015	0.297	12.6	36.8	27.3	0.06	5.01	11.2	33.28	41.06
Georgetown	0.750	0.617	0.017	0.283	10.9	35.5	26.5	0.08	5.39	13.5	32.31	33.46
Greenville	0.854	0.460	0.017	0.186	9.9	29.3	23.3	0.13	2.32	8.6	26.06	17.86
Greenwood	0.730	0.730	0.014	0.229	9.5	32.5	25.5	0.09	2.50	11.3	29.97	31.19
Hampton	0.768	1.105	0	0.288	15.6	43.5	32	0	7.35	12.9	38.65	53.41
Horry	1.140	0.612	0.033	0.199	9.8	27.6	22	0.10	2.48	11.9	32.08	13.28
Jasper	0.714	0.953	0.040	0.238	12.8	39.3	25.2	0.04	5.33	9.9	40.23	45.62
Kershaw	0.546	0.707	0.016	0.112	10.4	30.7	27.5	0.05	5.08	11.0	34.44	24.40
Lancaster	0.449	0.603	0.026	0.282	12	34.9	26.9	0.05	2.83	13.3	34.12	23.69
Laurens	0.421	0.737	0	0.180	13	38	32	0.05	6.49	11.6	36.02	25.26
Lee	0.422	0.474	0	0.263	13.4	41.5	31	0	8.57	16.2	38.31	64.07
Lexington	0.756	0.532	0.026	0.157	10.1	29.9	24	0.09	2.08	8.0	27.70	14.10
Mccormick	0.399	0.698	0	0.199	14.6	40.1	30.6	0	5.39	13.6	28.56	49.54
Marion	0.457	0.792	0.030	0.304	13.2	39.7	31.7	0.03	5.56	17.7	43.21	55.55
Marlboro	0.421	1.122	0	0.246	11.1	32.8	28.5	0	7.85	19.7	40.53	50.64
Newberry	0.477	0.901	0.027	0.159	12	34.7	26.5	0.05	4.24	10.3	32.04	30.75
Oconee	0.685	0.524	0.013	0.215	11	31.5	22.4	0.03	3.75	10.0	33.48	7.47
Orangeburg	0.751	0.947	0.011	0.239	14.3	41.4	27.8	0.07	5.90	15.8	35.43	61.93
Pickens	0.719	0.594	0.008	0.092	10.3	29.6	22	0.09	3.61	9.8	30.19	6.54
Richland	0.779	0.445	0.015	0.136	11.5	31.9	24.1	0.08	1.86	9.2	22.39	45.40
Saluda	0.251	0.704	0	0.151	11.8	33.2	27.6	0	5.94	9.7	40.59	26.14
Spartanburg	0.694	0.603	0.021	0.143	11.3	29.2	26.6	0.10	3.55	10.9	30.76	20.44
Sumter	0.521	0.661	0.009	0.186	13.8	34.6	29.8	0.06	4.09	12.0	30.60	46.63
Union	0.453	0.837	0	0.174	13.4	36	32.9	0.03	4.45	16.0	37.51	31.15
Williamsburg	0.381	0.763	0	0.235	15.3	43.1	31	0.03	11.57	16.7	40.02	65.44
York	0.711	0.486	0.022	0.174	9.1	27.9	21.6	0.09	2.31	11.0	28.18	18.85
South Carolina	0.59	0.69	0.014	0.19	12.14	34.95	27.59	0.05	4.81	12.56	33.85	35.95

### Bivariate maps

**Fig. 1:**
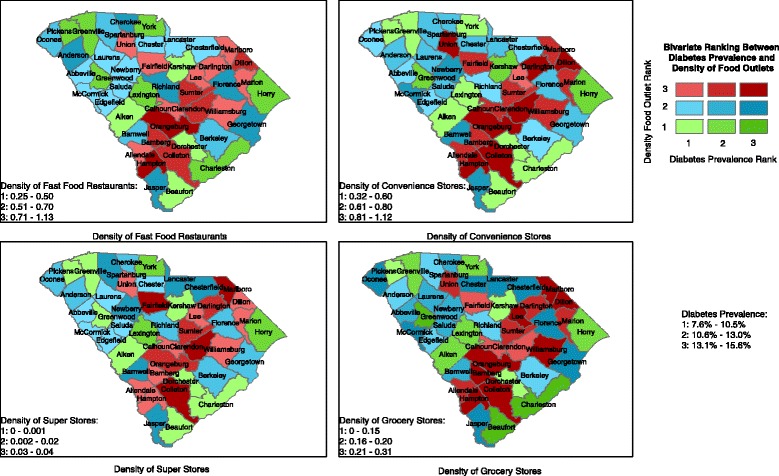
Bivariate ranking between Diabetes Mellitus prevalence and density of food outlets in South Carolina counties, 2011

Figure 1 displays the bivariate maps between the four food outlet densities and DM prevalence, each based on three ranks (i.e., low, medium, high). The highest rank combination formed between fast food restaurant densities and DM prevalence was observed in two PeeDee region counties: Hampton and Orangburg. The lowest rank combination was observed in Aiken, Beaufort, Dorchester, and Kershaw counties. The highest rank combination formed between convenience store densities and DM prevalence was observed primarily along the 1-95 corrdior (i.e., Clarendon, Colleton, Darlington, Dillion, Hampton, Orangeburg, and Union counties). The lowest rank combination was found in Aiken, Beaufort, Charleston, Dorchester, Greenville, Lexington, Pickens, and York counties. Rank combinations between super store density, grocery store density, and DM prevalence are also shown in Fig. 1.

### Correlations

Pearson correlation coefficients between each food outlet density and DM prevalence are presented in Table 2. Two food outlet densities reached statistical significance at the 0.05 level and were moderately correlated with DM prevalence: fast food restaurants (*r* = -0.45) and convenience stores (*r* = 0.54). Super stores (*r* = -0.21) and grocery stores (*r* = 0.16) had weak correlations with DM prevalence and did not reach statistical significance.

**Table 2:** Pearson’s correlation coefficients between density of food outlets and County-level diabetes prevalence in South Carolina, 2011

Food outlet	Correlation	*P*-value
Fast food restaurants	-0.45	0.007^a^
Convenience stores	0.54	<0.001^a^
Super stores	-0.21	0.16
Grocery stores	0.16	0.29

^a^Correlation is significant at the 0.05 level

*N* = 46

### OLS regression

Table 3 presents results from OLS regression analyses. Model 1 is a simple linear regression with each food outlet density considered as the only explanatory variable. The models predicted that for every 1 % increase in DM prevalence in a county, the density of fast food restaurants decreases by 4.52 % (*p*-value = 0.002), and the density of convenience stores increases by 5.33 % (*p*-value < 0.001). Model 2 is adjusted for the following county-level covariates: obesity, physical inactivity, recreation, unemployment, education, households with no car and limited access to a store, and race. The models show that 80 % of the variation in DM is explained by the variables (adjusted R-squared was consistent across all models). No food outlet density reached statistical significance in these adjusted models.

**Table 3:** Multiple-variable linear regression prediction County-level diabetes with density of food outlets in South Carolina, 2011

	Model 1			Model 2^b^		
Food outlet	Beta coefficient	Standard error	*P*-value	Beta coefficient	Standard error	*P*-value
Fast Food Restaurants	-4.52	1.34	0.002^a^	-0.55	0.90	0.54
Convenience Stores	5.33	1.24	<0.001^a^	0.89	0.86	0.31
Super Stores	-31.91	22.31	0.16	-0.40	11.66	0.97
Grocery Stores	4.61	4.30	0.29	-3.70	2.13	0.09

^a^Beta coefficients are significant at the 0.05 level

^b^Model 2 adjusted for obesity, physical inactivity, recreation facility density, unemployed, education, household with no cars and limited access to a store, and race

*N* = 46

## Discussion

Type II Diabetes Mellitus, along with other chronic diseases, is growing rapidly in South Carolina and the United States. Since the built environment has been shown to influence DM [13–15, 17, 21], this study examined the relationship between four types of food outlets accounting for density of population and the prevalence of DM. Both convenience stores and fast food restaurants are associated with poor food quality and thus were expected to be positively correlated with prevalence of DM. Likewise, super and grocery stores are associated with healthy food options and thus were expected to be negatively correlated with prevalence of DM. Pearson correlations revealed that the density of convenience stores had a moderate, positive correlation with DM, but the density of fast food restaurants had a moderate, negative correlation with DM. Super stores and grocery stores were not significantly correlated with DM prevalence.

The expectation that fast food restaurants and convenience stores are positively associated with DM and that super stores and grocery stores are negatively associated with DM was more evident in the bivariate maps. Aligned with our hypothesis, Hampton County for example, had the highest rank combination between fast food restaurant densities and DM prevalence as well as convenience store densities and DM prevalence. In fact, Hampton County has the highest prevalence of DM and obesity within South Carolina. This county also had the lowest rank of super store densities and the highest rank of grocery store densities. With Hampton County having one of the state’s smallest population densities, the lack of super stores is to be expected [31]. Small grocery stores are more common in rural counties [31].

Our results are unexpected due to the lack of healthful foods provided by fast food restaurants and convenience stores [20] as well as studies demonstrating the positive association between fast food restaurants and chronic diseases, such as obesity, at the macro level [13]. Studies found that proximity to fast food outlets is associated with greater food intake and availability of convenience stores is associated with increased risk of obesity [25]. Yet, studies have also been inconsistent with fast food restaurants resulting in negative or null associations between obesity and/or frequency of fast food consumption [30]. Jeffrey’s et al. study found no association between fast food proximity and fast food consumption or BMI [32]. The inconsistent findings in the literature may be caused by the lack of an established definition for fast food restaurants. This study used the definition based on NAICS code whereas other studies may provide their own definition. Additionally, the NAICS code is defined as food establishments where consumers pay before eating thus also including food establishments that provide healthy alternatives, such as Subway®. The inconsistent findings in the literature may also be due to differences in spatial scale. Ahern’s county-level study found obesity rates to be negatively associated with fast food restaurants and positively associated with grocery stores (in non-metro areas only), where as Maddock’s state-level analysis found obesity rates to be positively associated with fast food restaurants [13, 25].

The null findings regarding super store densities and grocery store densities to DM prevalence contradict the literature. Other studies found that larger food stores and super stores are associated with better access to high quality food, such as fresh fruits and vegetables [32–38], and the availability of supermarkets is associated with healthier diets, lower rates of obesity, and a longer life span [14, 23, 39–43].

The significant covariates in the regression models were obesity, physical inactivity, and race (except when considering the density of fast food restaurants as the exposure). This is expected because DM disproportionately affects non-Hispanic Blacks and African Americans. Previous studies have found that Blacks and disadvantaged groups are more likely to live in areas with inadequate access to healthy foods directly affecting their health [24].

### Limitations

This study has several limitations. DM, obesity, and physical inactivity were based on self-reported data collected by BRFSS. However, studies have shown self-report DM based on a physician’s diagnosis to be highly validated [44], and self-reported weight and height to compute BMI to also be validated among adults [45]. Additionally, BRFSS obtains county-level estimates by aggregating three years of data for a single estimate due to the limiting sample size preventing possible calculations for a single year. BRFSS also includes type I diabetes mellitus along with the DM data making it difficult to distinguish between the two types of diabetes; however, type II diabetes accounts for 90–95 % of all diabetes cases [11]. Furthermore, the study analysis did not consider spatial spillover: the impact of food outlets in bordering counties in other states. It is likely for individuals living in a border county to purchase food from nearby state counties. Moreover, because our total sample only included 46 counties, the power to detect statistical differences was reduced. Finally, the ecological nature of the study subjugates it to the ecological fallacy, thus limiting its inference to the county level only.

## Conclusions

Overall, findings from this ecological study do not show significant associations between any of the four types of food outlets and the prevalence of DM. This emphasizes the role of individual behavioral decisions on DM prevalence as well as the need for studies that examine the role of food outlet density on health outcomes at various spatial scales. DM and other chronic diseases are multi-faceted and relate to many factors including both individual and environmental as well as social and cultural. Therefore, to further understand the impact on DM, future research should include both the structure of the built environment- food outlets and recreational space- and individual factors in order to construct a thorough, comprehensive model of the contextual factors contributing to the increase of chronic diseases. Assessing the environmental, social, and cultural determinants of chronic diseases is key in establishing health-promoting environments. There is a strong need to advocate for such environmental change.

## Additional file

Additional file 1:
**Study Variables’ Data Sources.** (DOC 35 kb)
