# Supplementary material for: An ecological analysis of food outlet density and prevalence of type II diabetes in South Carolina counties
Source: BMC Public Health. 2016 Jan 5;16:10. doi: 10.1186/s12889-015-2681-6 (PMC4700568; doi:10.1186/s12889-015-2681-6)
Supplement: Additional file 1: — Study Variables’ Data Sources. (DOC 35 kb) [file 12889_2015_2681_MOESM1_ESM.doc]

# Additional files

### Additional file 1 – Study Variables’ Data Sources

| **Variables** | **Definition** | **Source** |
| --- | --- | --- |
| Diabetes | Age-adjusted percentage of adults (age ≥ 20) in South Carolina with diabetes excluding those with gestational diabetes in 2011 | Centers for Disease and Control, 2011 |
| Fast Food Restaurant Density | Number of limited-service restaurants in each county in South Carolina per 1,000 residents in 2011 | US Department of Agriculture Economic Research Service, 2011 |
| Convenience Store Density | Number of convenience stores in each county in South Carolina per 1,000 residents in 2011 |
| Super Store Density | Number of supercenters and warehouse clubs in each county in South Carolina per 1,000 residents in 2011 |
| Grocery Store Density | Number of supermarkets and grocery stores in each county in South Carolina per 1,000 residents in 2011 |
| Obesity | age-adjusted percentage of adults (age ≥ 20) in South Carolina with a body mass index ≥ 30 in 2011 | Centers for Disease and Control, 2011 |
| Physical Inactivity | Age-adjusted prevalence of adults (age ≥ 20) in South Carolina, who reported no leisure-time physical activity in the past 30 days in 2011 |
| Recreation | Number of recreational facilities in each county in South Carolina per 1,000 residents in 2011 | US Department of Agriculture Economic Research Service, 2011 |
| Unemployment | Unemployment rate in each county in South Carolina in 2011 | US Census, 2010 collected from Bureau of Labor Statistics, 2011 |
| Education | Percentage of adults with only a high school diploma from 2009 – 2013 in each county in South Carolina | US Census, 2010 collected from American Community Survey 2009 – 2013 |
| Households with no car and limited access to a store | Percentage of housing units in each county in South Carolina with no car and at least one mile away from a supermarket or large grocery store in 2010 | US Department of Agriculture Economic Research Service, 2011 |
| Race | Percentage of each county’s resident population in South Carolina that is non-Hispanic Black or African American in 2010 | US Department of Agriculture Economic Research Service, 2011 |
